# Level of knowledge, skills and attitude of trainee teachers on Web 2.0 applications in teaching geography in Malaysia schools

**DOI:** 10.1016/j.heliyon.2021.e08568

**Published:** 2021-12-08

**Authors:** Fauziah Che Leh, Arnold Anduroh, Miftachul Huda

**Affiliations:** Faculty of Human Sciences, Universiti Pendidikan Sultan Idris, Malaysia

**Keywords:** Geography trainee teacher, Knowledge, Skills, Attitude, Geography teaching, Web 2.0

## Abstract

This study aims to discuss the level of knowledge, skills and attitude of trainee teachers from the Geography and Environment Education Program in Universiti Pendidikan Sultan Idris on the use and effectiveness of Web 2.0 applications in the teaching and learning process of the Geography subject in Malaysia schools. This research applied purposive sampling by using a questionnaire as a primary data collection and distributed to the respondent by Google Form. 100 trainee teachers undergoing teaching training sessions in various schools in Malaysia were selected as the study sample and the data collection was carried out from April to June 2020. By using SPSS, descriptive analyses such as frequency, percentage, mean and standard deviation were used to analyse and present the data. The findings of the study showed that the mean values for the level of knowledge (mean = 4.09), skills (mean = 3.69) and attitude (mean = 3.98) are high. We have proposed some recommendations to the Universiti Pendidikan Sultan Idris (UPSI) and Trainee Teachers of the Education Program to further reinforce the implementation of integrating Web 2.0 applications into the teaching and learning of geography. In implications, this study can guide trainee teachers and other stakeholders to consider the teaching strategies that are appropriate for the use of Web 2.0 applications in the classroom. The selection of a Web 2.0 application design that fits to the learning objectives can encourage the teaching and learning (T&L) process and subsequently helps in the improvement of students' academic achievement in the future.

## 1 Introduction

In recent decades, the advancement of science and technology have contributed to the creation of various innovations in new fields of education (Uche et al., 2016; Exarchou et al., 2015). This progress is in line with the changing times that are taking place among today's generation that has the potential to develop ideal talent at the global level. The approach of using Web 2.0 applications in the teaching and learning process can allow students the opportunity to explore the learning methods that can lead to the collaboration of information access and information sharing. As such, the extent of Web 2.0 applications could improve the mastery of 21st century skills such as collaboration skills, information access skills and online learning (Aminin et al., 2018; Ellis et al., 2020; Maseleno et al., 2018a). In this regard, each student will be able to gather, collect and display any existing or newly explored information to maximize the use of technology in teaching and learning activities, in and out of the classroom. Moreover, the essence of Web 2.0 applications play a role as an agent in processing, managing, manipulating, storing, transferring and disseminating information through electronic media (Hursen, 2020). Therefore, the teaching and learning (T&L) process does not only occur during school hours but in fact, users can carry it out anywhere through online learning without being restricted to a certain time and place.

Web 2.0 applications are used as a study in the teaching and learning process in geography subject in Malaysia school because cyberspace is the lifeblood of human life, especially the younger generation through services and business using the Internet as an intermediary, especially during the Covid-19 pandemic. This is because Web 2.0 allows students the opportunity to explore the learning methods that can lead to the collaboration of information access and information sharing during pandemic Covid-19. Web 2.0 applications also can improve the mastery of 21st century skills such as collaboration skills, information access skills and online learning in a situation where face to face teaching and learning cannot be carried out (Khalili et al., 2021; Moksin et al., 2018; Mulyadi et al., 2022). In this regard, Web 2.0 recently relevant to the education system especially during the post-pandemic which allows each student will be able to gather, collect and display any existing or newly explored information to maximize the use of technology in teaching and learning activities, in and out of the classroom.

Furthermore, the use of Web 2.0 applications has the potential to help teachers and students create an effective learning and teaching environment and facilitate mixed learning in the classroom to run more effectively. This is because, a conducive, effective and efficient T&L environment will encourage intellectual, friendship, cooperation and support activities as well as assist students in the aspects of physical, emotional, spiritual, and intellectual development and the students' learning itself. In particular, the atmosphere circumstance could influence students' social, psychological and pedagogical aspects (Bahrudin et al., 2021; Gamji et al., 2021; Maseleno et al., 2018b). As a result, Web 2.0 applications are not only developed as a teaching aid for teachers in the classroom but their use is more towards building quality human capital for the technological environment (Isaias et al., 2019; Othman et al., 2016). Overall, Web 2.0 applications help in the teaching and learning process of students and teachers especially in project-based learning and problem-based learning activities. This is because the tools available in Web 2.0 have opened up more space and opportunities in increasing student involvement in the T&L process in the classroom.

In addition, the learning approach in the use of Web 2.0 applications is in line with the current national education systems. In addition to maximizing Information Communication Technology (ICT) in T&L, it can also help students to increase their interest in mastering geography subjects in school. However, studies on the use of Web 2.0 applications in teaching and learning (T&L) have yet to be conducted more widely, especially on geography subjects in schools. This is due to some constraints faced by teachers resulting in them not being able to integrate the Internet and ICT into T&L. In a report by (UNESCO (2013), it is stated that 80% of teachers in schools spend less than an hour a week using ICT in the classroom. With this regard, the use of smartphone in schools for instance is becoming essential to value on the learning support (Anshari et al., 2017). In order to achieve the balance, educators are urged to equip themselves with various knowledge and techniques or skills based on information technology to face the challenging teaching world. In this regard, educators need to be prepared with sufficient knowledge and skills in the use of ICT. This is because one day students may go to school not only carrying books but also carrying laptops as a replacement for books.

However, there has been less scholarly attention to look into detail on knowledge, skills, attitude of geography teachers, especially those that involve trainee teachers through the use of Web 2.0 applications. It is focused on the particular overview in the context of teaching on Geography in Malaysia. As such, this article aims to discuss trainee teachers' acceptance of Web 2.0 applications by identifying the level of knowledge, skills and attitude to develop Web 2.0 applications to support the 21st century learning in the teaching and learning process today. This has already happened in developed countries. Researchers have been encouraged to research the level of knowledge, skills and attitude regarding the use of Web 2.0 applications and their effectiveness in the teaching of geography in schools. Through this study, the advantages and disadvantages of trainee teachers can be scientifically investigated for future use.

## 2 Literature review

### 2.1 Web 2.0 digital application on teaching education

Web 2.0 applications result from the development of a complex Internet into something more dynamic and flexible. The use of Web 2.0 applications in teaching and learning creates a more effective environment, in the sense that the approach of using Web 2.0 can shape the way students learn, the way teachers teach and the way teachers interact with students (Bal-Taştan et al., 2018). Its significance in education could be viewed into teaching technology support in encouraging informal discussions, dialogue, collaborate and share knowledge openly. The particular emphasis could be given into the opportunity in enabling the students to choose teaching aids with its important element to increase their motivation (Maseleno et al., 2018c; Morales and Calvo, 2017). For instance, the use of YouTube which can improve proficiency in English speaking skills, grammar and writing skills (Caliskan et al., 2019), where its beneficial value could be more elaborated into improving the understanding of learning English (Lv and Luo, 2021), and even in improving Arabic language skills (Rahil and Harun, 2016).

In addition, the use of Blog applications is widely promoted in order to improve understanding, writing and learning skills. Moreover, the significance could be viewed to improve students' understanding of the subject of Basic Economics (Fırat and Köksal, 2017), to enhance the positive value on vocabulary, discourse markers, writing organization and writing structure (Almekhlafi and Abulibdeh, 2018), and to improve reading, writing and collaborative skills among students (Rizou and Klonari, 2020). In particular, its application at a continued commitment could improve reading skills, writing and interaction skills as well as save time. The use of the Facebook application is also widely applied in T&L, which becomes a suitable medium for learning, supporting interactions between peers and conversations regarding course materials (Alan, 2017). The mutual line of this benefit could be viewed further through the use of “Facebook Group” in enhancing the enthusiasms in raising their way of learning, and also in increasing an active discussion practices in online basis (Fearnley, 2020). Thus, it can be concluded that Facebook applications can encourage collaboration among teachers or students.

In further, the use of the Twitter application in the education setting could enhance the positive value towards learners' rational and critical thinking (Mukherjee, 2019; Sukadari and Huda, 2021). The particular point is that the students showed a positive attitude towards the use of Twitter throughout their studies (Bista, 2015). Moreover, the use of Twitter is also able to improve reading skills, especially in English Fun Learning (EFL) (Mompean and Fouz-González, 2016). It is similar that the use of Twitter may also improve the mastery of English speaking skills, grammar and writing skills (Chang and Wu, 2018). When the use of the Prezi application was also applied in education setting, its continued application could give an insight into enhancing the experience, especially in conducting teaching and learning in the classroom (Duffy et al., 2015). Moreover, the use of Prezi could help improve the vocabulary memorising skills among students (Wafaa, 2015). It indicates that the use of Web 2.0 applications can affect English reading skills performance (Al-Hammouri, 2018). Apart from improving reading skills, the use of the Prezi application can also attract students' interest in education setting (Musdalifah, 2019).

### 2.2 Technology Acceptance Model (TAM)-based application on teaching education

**Figure 1:**
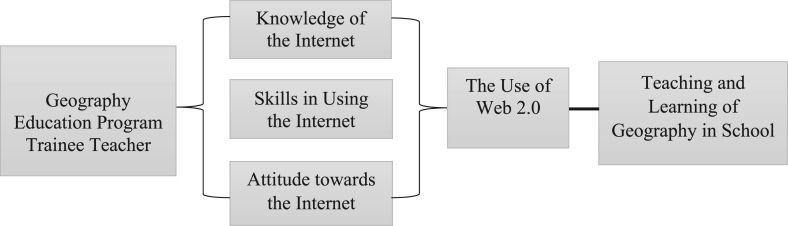
Conceptual framework (source: modified from Rohani, 2011).

The research framework was built based on the Technology Acceptance Model by Davis et al. (1989). It states that if a person has a good level of skills and minimum constraints in the use of the Internet it can be used as a learning medium in schools (Bauder, 2017). The study framework is as shown in Figure 1. This attitude in turn leads to intention, which is then translated in the form of behaviour. The Technology Acceptance Model describes user acceptance towards the use of technology, especially information systems. This model states that the actual use of a system, such as a computer-based system, is determined by the user's attitude towards the system. The actual use of a system can be measured through the users' knowledge and skills of the system (Teo et al., 2019).

Previous studies have shown that the main constraint faced by teachers in conducting teaching in the form of Web 2.0 applications was the lack of preparation by teachers in handling ICT. The detail could be viewed that teachers still had a medium level of knowledge and skills in using ICT (Feldman and Sanger, 2007). In particular, the lack of exposure to Web 2.0 applications in the T&L process causes teachers' preparedness to end up being less encouraging (Bal-Taştan et al., 2018), and thus preschool teachers need exposure to computer-related courses to get them prepared for the use of ICT (Morales and Calvo, 2017). Exposure to computers, especially on Web 2.0 applications, is very important to ensure that the T&L process is in line with today's growing development of technology.

In addition, there was stated that less attention amongst secondary school teachers have never used a computer either in school or out of school, due to the lack of self-confidence in using computers (Caliskan et al., 2019). Describing the reason for the negative attitude towards computers was due to the levels of knowledge and experience in the field. Furthermore, the teachers' less conviction to the traditional teaching methods is required to adapt to the instructional technology (Lv and Luo, 2021), where most experienced teachers have a mind-set of the teaching methods that they like (Fırat and Köksal, 2017). The ability of Web 2.0 applications to support, encourage informal discussions, dialogue, collaboration and share knowledge openly can benefit students. However, the lack of Web 2.0 applications in teaching and learning (T&L) for the Geography subject causes students to not be able to fully maximize the use of the Internet. As a result, the lack of knowledge and skills in the use of technology was the reason for the failure of ICT integration in T&L (Almekhlafi and Abulibdeh, 2018). In particular, the teacher's useless technology in T&L in the classroom comes from the lack of knowledge and skills in using ICT, in the sense that teachers do not make use of the advantages of ICT in T&L. Therefore, teachers' preparedness is very important in facing changes in education, especially on the use of ICT in T&L.

## 3 Research methodology

This research applied purposive sampling as a survey method by using a questionnaire for primary data collection and distributed to the respondent by Google Form due to pandemic Covid-19. The study sample comprised 100 trainee teachers from the Geography Education Program who had undergone teaching training in schools (level 2) and the data collection was carried out from April to June 2020. The questionnaire containing five sections, namely Section A on respondents' information, Section B on levels of knowledge on the use of Web 2.0, Section C on levels of skills in using Web 2.0, Section D on attitude towards the use of Web 2.0, Section E on levels of use of Web 2.0 applications and Section F on the effectiveness of the use of Web 2.0 in teaching Geography in schools. The items developed in this questionnaire are adapted from a study which are aligned with the problem statement and objectives of the study (Embi, 2011). All items were tested through a pilot study item validation. The levels of validity and reliability of the items were tested using Cronbach's alpha coefficient. By using SPSS, descriptive analyses such as frequency, percentage, mean and standard deviation were used to analyze and present the data. The results of the analysis showed that the value of the reliability coefficient for the whole data was high, which is 0.838. Therefore, the item statements could be used among the actual study samples.

## 4 Findings

The background of the respondents based on the age factor, it was found that the respondents have only consisted of 23–25 years old age group as shown in Table 1.

**Table 1:** Respondents' background by race factor.

Race	Frequency (*f*)	Percentage (%)
Malay	54	q54
Chinese	3	3
India	1	1
Bumiputera Sabah	26	26
Bumiputera Sarawak	14	14
Others	2	2

**Total**	**100**	**100**

### 4.1 Level of knowledge of the use of Web 2.0 among trainee teachers

Table 2 shows the mean score for trainee teachers on the knowledge of the use of Web 2.0 concerning about measuring scale (Feldman and Sanger, 2007). In Table 2, it can be seen that the highest mean score for trainee teachers' knowledge of Web 2.0 is on the item *“I can use Google platform to surf the Internet”* with a mean value of 4.72 and a standard deviation of 0.494. While the lowest mean value is of the item *“I can use various methods in Web applications for T&L purposes”* with a mean of 3.33 and a standard deviation of 0.779. Overall, it was found that the items for the trainee teacher's level of knowledge were at a high level with a mean value of 4.09 and a standard deviation value of 0.582.

**Table 2:** Level of knowledge of the use of Web 2.0 among trainee teachers.

Item	Mean	Standard Deviation	Level of Knowledge
1. I can use the Google platform to surf the Internet	4.72	0.494	High
2. I know how to use e-mail as a medium for sharing information with students	4.70	0.502	High
3. I can download and upload information for T&L purposes	4.70	0.460	High
4. I can use various methods in Web applications for T&L purposes	3.33	0.779	Medium
5. I know how to adapt the use of materials to diversify R&D activities in the classroom	4.42	0.589	Medium
6. I often improve my knowledge and skills through new technology learning opportunities	3.64	0.630	High
7. I can design and build (individually or in groups) integrated learning activities through Web applications in and out of the classroom	3.41	0.604	Medium
8. I can develop a variety of application media on the Web for students' references	3.37	0.544	Medium
9. I know how to sign in and sign out any Web application that I access	4.57	0.536	High
10. I can access YouTube for T&L purposes	4.68	0.510	High
11. I ​know how to use all applications on the Web	3.49	0.758	Medium

**Total Sum**	**4.09**	**0.582**	**High**

### 4.2 Level of skills in the use of Web 2.0 among trainee teachers

Table 3 shows the mean scores for the level of skills in the use of Web 2.0 among trainee teachers. The measurement scale for this test was based on the measurement test (Feldman and Sanger, 2007). Based on Table 3, the highest mean for teachers' perception towards the level of skills in the use of Web 2.0 is in the item *“I am capable of using Google to surf the Internet”* with a mean value of 4.68 and a standard deviation of 0.489, followed by the item *“I always use information from the Internet Websites”* with a mean value of 4.63 and 0.525 is the standard deviation. The item *“I assist in updating the school Website”* recorded the lowest mean with a mean value of 2.22 and a standard deviation of 0.785, followed by 2.47 the second-lowest mean value for the item *“The Web application I use for T&L is always updated to ensure new information”* and its standard deviation is 0.501. Overall, it was found that the level of skills in the use of Web 2.0 among trainee teachers is at a high level with a mean value of 3.69 and a standard deviation of 0.637.

**Table 3:** Level of skills in the use of Web 2.0 among trainee teachers.

Item	Mean	Standard Deviation	Level of Skills
1. I can use the Website platform as a search medium	4.55	0.500	High
2. I always use information from Internet Websites	4.63	0.525	High
3. I am capable of using Google to surf the Internet	4.68	0.489	High
4. Educational Websites help me a lot in getting information	3.20	0.816	Medium
5. I have extensive skills and knowledge in using the Web	3.34	0.755	Medium
6. I can use Web applications to download and upload information for T&L purposes	3.51	0.703	High
7. The Web application I use for T&L is always updated to ensure new information for students	2.47	0.501	Low
8. I can create and develop videos and graphics through Web applications	4.26	0.690	High
9. I can communicate with students through social applications	4.34	0.713	High
10. I can make Slideshare for T&L purposes in and out of the classroom	3.45	0.538	Medium
11. I assist in updating the school Website	2.22	0.785	Low

**Total Sum**	**3.69**	**0.637**	**High**

### 4.3 Attitude towards the use of Web 2.0 among trainee teachers

Table 4 shows the level of attitude towards the use of Web 2.0 among trainee teachers in teaching in schools. There are 11 items in this section based on the questionnaire. In this section, there are three levels of teacher's attitude towards the use of Web 2.0, namely low level, medium level and high level (Feldman and Sanger, 2007). Based on Table 4, the highest mean value is in the item *“WhatsApp can be a very good communication tool for everyone”* with a mean value of 4.70 equivalents to the standard deviation of 0.482. For the medium level, the item *“Web applications can replace textbooks in the classroom”* is the lowest with a mean value of 2.76 and a standard deviation of 0.954. The item *“using Web applications is a waste of time”* is the second-lowest item for the medium level with a mean of 2.87 equivalents to the standard deviation of 1.404. However, the overall level of attitude towards the use of Web 2.0 is high with a mean of 3.98 and a standard deviation of 0.729.

**Table 4:** Attitude towards the use of Web 2.0 among trainee teachers.

Item	Mean	Standard Deviation	Level of Attitude
1. Facebook, Blog and Twitter are the main media for ​people to share information and opinions	4.50	0.643	High
2. WhatsApp is great to be used as a communication tool by everyone	4.70	0.482	High
3. I learn new technologies easily	3.47	0.673	Medium
4. The information I find in Web applications is not necessarily accurate, especially other people's blogs	4.53	0.610	High
5. Educational Web Portals ​help to improve my learning	4.42	0.589	High
6. Web applications are interesting media in the classroom	4.50	0.559	High
7. Most of the Web technologies I know can improve T&L strategies as well as improve students' ability to think	4.48	0.521	High
8. Web applications can replace textbooks in the classroom	2.76	0.954	Medium
9. I am not sure about the features ​available in Web 2.0 applications	3.41	0.933	Medium
10. The use of Web applications is a waste of time	2.87	1.404	Medium
11. I am responsible for operating Web applications for T&L purposes	4.18	0.657	High

**Total Sum**	**3.98**	**0.729**	**High**

### 4.4 Assessment of trainee teachers' level of use of Web 2.0 applications

Table 5 shows the level of use of Web 2.0 among Geography education program trainee teachers. In the questionnaire items, there were 20 types of Web 2.0 applications measured based on three Likert scales, namely have never known about the application, know but not using the application, and have been using the application. The extent of measurement scale for mean (Feldman and Sanger, 2007), researchers were able to observe the level of teachers' knowledge of Web 2.0 applications. Overall, the level of use of Web 2.0 applications among trainee teachers was at a low level. This indicates that the trainee teachers of the Geography education program had not been using Web 2.0 applications. The total mean score for this level is 1.92 and the standard deviation is 0.531. There were three medium levels of teachers using these applications, namely Slideshare, Facebook and Prezi. However, Facebook showed the highest mean of 2.69 and a standard deviation of 0.464. The lowest level of use of Web 2.0 was Del.Icio. Us with a mean of 1.16 and a standard deviation of 0.394. However, there was a high level of trainee teachers on the use of Web 2.0 applications, which is YouTube, with a mean value of 3.00 with a standard deviation of 0.00.

**Table 5:** Assessment of trainee teachers' level of use of Web 2.0

Item	Mean	Standard Deviation	Level of the Use of Web 2.0
1. Blog	2.25	0.457	Low
2. Diigo	1.27	0.468	Low
3. Elluminate	1.39	0.584	Low
4. Evernote	1.47	0.610	Low
5. Facebook	2.69	0.464	Medium
6. Flickr	2.44	0.608	Low
7. Glogster	1.50	0.522	Low
8. Jing	1.69	0.614	Low
9. Ning	1.67	0.603	Low
10. Prezi	2.57	0.555	Medium
11. Skype	2.13	0.485	Low
12. Slideshare	2.64	0.502	Medium
13. Twitter	2.42	0.589	Low
14. Voicethread	1.45	0.575	Low
15. Wallwisher	1.36	0.541	Low
16. Wiki	2.49	0.658	Low
17. YouTube	3.00	0.000	High
18. Wordle	1.36	0.611	Low
19. WordPress	1.55	0.783	Low
20. Del.Icio.Us	1.16	0.394	Low

**Total Sum**	**1.92**	**0.531**	**Low**

### 4.5 The use of Web 2.0 applications in teaching and learning (T&L)

Table 6 shows the frequency and percentage of use of Web 2.0 applications in the teaching and learning (T&L) of Geography in schools. The results of the study showed that all of them had been using one of the Web 2.0 applications in teaching and learning (T&L). Among the types of Web 2.0 applications used by the trainee teachers were YouTube, Slideshare, Prezi, and Facebook. This shows that the trainee teachers from the Geography education program have applied modern teaching and limited the amount of traditional teaching in the T&L in schools.

**Table 6:** The use of Web 2.0 applications in teaching and learning (T&L).

Item		Frequency (*f*)	Per cent (%)
The Use of Web 2.0 applications	Yes	100	100
	No	0	0

**Total**		**100**	**100**

### 4.6 The effectiveness of using Web 2.0 in T&L among trainee teachers

A descriptive analysis was performed to evaluate the efficiency of using Web 2.0 applications in T&L among Malay language teachers. Eleven items were given to the study respondents and based on Table 7, the item with the highest mean score is *“textbook content, library and trainee teachers' knowledge can be enhanced with the new approach of using Web 2.0 applications”* with a mean value of 4.55 and a standard deviation of 0.557 at a high level. The lowest mean score is *“the use of the Internet can meet the various needs of students separately through trainee teachers' method of teaching and learning (T&L)”* with a mean value of 4.29 and a standard deviation of 0.700. Even though the mean score is the lowest, the level of effectiveness is still at a high level. Overall, the level of effectiveness of using Web 2.0 applications in T&L among trainee teachers is at a high level with an overall mean score of 4.45 and a standard deviation of 0.623.

**Table 7:** The effectiveness of using Web 2.0 applications in T&L among trainee teachers.

Item	Mean	Standard Deviation	Level of Knowledge
1. I get to upload teaching and learning materials easily, interestingly and effectively for students	4.53	0.521	High
2. Can increase my knowledge of Internet use and computer literacy	4.52	0.521	High
3. The use of Web 2.0 applications can increase students' interest in T&L	4.53	0.610	High
4. The Internet provides up-to-date information on various related topics that are ​not available in other sources	4.37	0.645	High
5. Textbook content, library and knowledge of trainee teachers can be enhanced with the new approach to the use of Web 2.0 applications	4.55	0.557	High
6. Learning is more student-centred through an application-based approach in Web 2.0	4.38	0.693	High
7. Knowledge in the use of Web applications can improve communication skills	4.36	0.718	High
8. The use of the Internet can meet the various needs of students separately through trainee teachers' method of teaching and learning (T&L)	4.29	0.700	High
9. Web 2.0 software is an intermediary tool that is not influenced by race, culture and sex	4.45	0.701	High
10. The use of Facebook, blogs, video sharing (YT), Twitter and Slideshare can encourage online learning	4.53	0.593	High
11. I get to develop skills through new experiences gained from using Web 2.0	4.51	0.594	High

**Total Sum**	**4.45**	**0.623**	**High**

The results of the quantitative analysis in this study showed that most of the trainee teachers had a high level of knowledge, high level of skills, high level of attitude, low level of use of Web 2.0 and level of effectiveness of using Web 2.0 applications in T&L among trainee teachers.

### 4.7 Trainee teachers' level of knowledge on the use of Web 2.0 applications in teaching in schools

This study showed that the level of knowledge of trainee teachers on the use of the Internet as a whole was at a high level. The overall mean for the trainee teachers' knowledge level was 4.09 with a standard deviation value of 0.582. This finding is supported by a study conducted by Idris and Adam (2010) on the use of the Internet among primary school teachers in Johor. The research conducted by the researcher included basic knowledge in the use of the Internet. The researcher found that teachers mastered the basic knowledge of Internet use with a percentage rate of 83.5 per cent of the 132 teachers in the study. This percentage rate indicates that current teachers, including trainee teachers, have basic knowledge in using the Internet. On the other hand, a study has been conducted by Rozaidi (2000) on basic knowledge of Internet use among secondary school teachers in Johor Bharu. The researcher's analysis found that teachers' basic knowledge of Internet use was at a low level with a percentage of only 22.4%. This proves that throughout the year, there has been an increase in Internet use among teachers in Malaysia.

### 4.8 Trainee teachers' level of skills on using Web 2.0 applications in teaching in schools

These findings indicate that the level of skills of trainee teachers on Internet use in teaching in schools was at a high level with a mean value of 3.69 and a standard deviation of 0.637. This study is also supported by Rosnaini et al. (2011) regarding the level of integration of ICT among smart boarding school teachers at the Institute of Teacher Education, Tengku Ampuan Afzan Campus, Pahang. The study conducted covered the level of basic ICT skills. From the researcher's finding, the teachers' level of basic skills in ICT was high with a mean value of 3.13 and a standard deviation of 0.54. Meanwhile, a study conducted by Idris and Adam (2010) among primary school teachers around Johor Bahru showed that teachers had basic skills in using the Internet with a percentage of 76.66%. This shows that teachers have basic skills in using the Internet.

### 4.9 The attitude of geography education program trainee teachers towards the use of the Internet in teaching in schools

This study found that the attitude of trainee teachers towards the use of the Internet in teaching geography in schools was high with a mean of 3.98 and a standard deviation of 0.729. A study was conducted by Ching and Badusah (2010) on the use of ICT in the teaching among Malay language teachers in 12 primary schools in Bintulu, Sarawak. The scope of the researchers' study included teachers' attitudes towards the use of ICT. The researchers found that teachers' attitude towards the use of ICT in teaching in schools was high with a mean score of 3.74. This analysis found that teachers in 12 schools showed a positive level in the use of ICT in teaching in schools. A study by Naser et al. (2010) on the attitude of rural school teachers in Jordan towards ICT among teachers' attitude towards ICT showed that it was at a high level with a mean of 3.19 and a standard deviation of 1.43. However, Naser et al. (2010) also found that attitude towards the use of ICT among teachers in rural areas in Jordan was at a low level with a mean value of 2.52 and a standard deviation of 1.19. Furthermore, a study has been conducted by Baharuddin and Badusah (2016) on the use of the Internet among Malay language teachers in Kuala Langat. The researchers have highlighted issues related to the attitude of Malay language teachers towards the use of the Internet. The results of the study showed that the attitude of Malay language teachers in Kuala Langat towards the use of the Internet was at a medium level.

## 5 Analysis and discussion

Most trainee teachers showed a positive level on the use of Web 2.0 in the teaching and learning of Geography in schools in Malaysia. However, the variables of skills (mean = 3.69) are lower compared to knowledge (mean = 4.09) and attitude (mean = 3.98). This means teachers need to upgrade the skills of using Web 2.0 during the T&L session of geography subject in school. Geography trainee teachers and other trainee teachers in Malaysia are different in the context of knowledge, skill and attitude, level of use and level of assessment on the effectiveness of T&L via ICT application. Geography trainee teachers expose to ICT through university curriculum is formed in terms of geography knowledge and skills together with pedagogical methods based ICT (Rizou and Klonari, 2020). This gives an advantage because geography students will eventually go to secondary school to teach geography subjects exposed to a technical subject syllabus from an early stage. A comparison between geography teachers in Malaysia and throughout the world will show a different scenario (Fearnley, 2020). This is because this study focuses on geography trainee teacher as a study subject, not geography teacher who teaches in a real school. So the experience of using ICT is different because they are assessed during a teaching training session for 14 weeks. Furthermore, the training of teaching them runs in urban and rural schools which affects Internet access as an important basis in determining the success of this VLE.

In addition, the value of this study might give a strategic pathway to the government's support and efforts in realizing the concept of virtual learning through the introduction of various T&L mediums. This aims to improve performance and respond to the call towards a developed country. Nevertheless, the technical challenges are also undeniable as it is a key resource for accessing e-learning (Mukherjee, 2019). The International Study of Learning (TALIS) reports that 53% of schools in Malaysia still do not have adequate computer facilities, 57% of schools do not have Internet access, and 41% of schools still lack teaching aids for use in the classroom (Hapini, 2019). Although teachers' acceptance of Web 2.0 was positive, problems in terms of technical support hampered its implementation. E-learning programs can help improve the standard of education but it depends on the provision of complete technical facilities (Collins and Halverson, 2009). Inadequate technical support for educators and students to use ICT during teaching and learning makes the situation unconducive for learning (Chang and Wu, 2018). This will cause teachers to choose to teach conventionally as facilities such as computers and Internet access are limited. This is often experienced by most rural schools that do not have adequate Internet access and computer equipment.

Previous experience has shown that Internet access, technical support, lack of effective training, limited time and lack of teacher competence are among the factors that the use of VLE in Malaysian rural schools does not reach a satisfactory level. Four suggestions to increase the level of use and mastery of ICT include organizing programs to improve skills in the use of ICT by involving skilled and competent teachers as instructors (Uche et al., 2016). This is followed by establishing a policy that requires teachers to acquire ICT skills before being appointed as teachers. In this view, the Ministry should play a role by creating a more transformative professional development program by focusing on 21st century teaching skills for teachers. Moreover, the school administration must ensure that all teachers attend seminars, training, and workshops on the use of ICT organized by the Ministry of Education. As a result, the active participation of teachers in e-skills related programs provided by the ministry can increase the awareness and knowledge of teachers in the 21st century. Through the implementation of this program as well, all the feedback related to e-learning can be collected to be evaluated for improvement in the implementation strategy in the future (Ghavifekr et al., 2012).

### 5.1 Assessment on the level of use of Web 2.0 between geography teachers and other teachers in Malaysia

Overall, it was found that the level of use of Web 2.0 among trainee teachers in the teaching of Geography in schools was at a low level with a mean value of 1.92 and a standard deviation of 0.531. A study conducted by Baharuddin and Badusah (2016) also showed that the level of knowledge of Web 2.0 among Malay language teachers was at a medium level with a mean score of 1.60 and 0.515. The study has been conducted by Norazlinda and Surendran (2014) on the use of Web 2.0 tools among university lecturers. The study was conducted to find out the lecturers' level of knowledge on the use of Web 2.0 tools in teaching and learning (T&L) at the University. The analysis of the study found that the level of knowledge of Web 2.0 was at a medium level with a mean of 3.92 and a standard deviation of 0.92. Based on the results of the study on lecturers' level of knowledge, it can be concluded that the constraint faced by school teachers is also due to the lack of exposure to the use of Web 2.0 from the university lecturers.

However, the role of the educator is important in building a more inclusive combination of teaching and learning for all students through the use of Web 2.0. The study analysis showed that there were several Web 2.0 applications used by trainee teachers in teaching and learning (T&L) such as YouTube, Slideshare, Prezi, and Facebook with a medium and high mean score. Based on the application used by trainee teachers, it can be concluded that all of the teaching and learning (T&L) methods applied were in the form of listening (Audio), seeing (Visual) and doing (Kinesthetic). Thus, this study is in line with Lindstrom (1994) who stated that 75% of students understood better when they could hear, see and do work compared to those who only see and hear (40%) as well as see (20%).

The use of YouTube applications in Teaching and Learning (T&L) can improve the skills in a particular subject. For instance, a study by Rahil and Harun (2016) found that YouTube could improve listening skills. The researchers' study has shown the level of use of YouTube application in Arabic language listening skills among Higher Learning Institute (HLI) in Malaysia. The analysis of the study showed that a total of 53.3% of students agreed that they were using the YouTube application in learning Arabic. Students also agreed that their Arabic vocabulary listening skills were improved by watching videos from YouTube. As a result of the analysis made by the researchers, more students agreed (46.7%) as compared to 10% of students who disagreed that YouTube helped them in improving their Arabic vocabulary.

In addition, the integration of technology in T&L can also improve communication between teachers and students as well as students and students. This is shown by the use of Web 2.0 applications which is a favourite trend of society today, including educators and students. Web 2.0 applications could be more open and easy to be used by everyone. Web 2.0 applications are also able to strengthen relationships with students through the sharing of information and the communication held in social networks (Amiruddin et al., 2020). Therefore, the social networks in the 2.0 application can bridge the gap as well as assist in teaching and learning (T&L).

Next, the acceptance of a positive attitude towards ICT can also be found in previous studies, such as in the teaching of science and mathematics or other subjects. This study can also be seen from the use of ICT in the subject of Humanities Stream and Technical and Vocational Stream (Amiruddin et al., 2020). Moreover, the detailed point could be viewed into the use of ICT for the subject of Economics and also language studies (Emrah and Selami, 2015). Based on the studies conducted, it can be concluded that the acceptance towards the use of ICT in teaching and learning shows a positive attitude. Thus, it is also found in this study that trainee teachers showed a positive attitude towards the use of ICT in teaching Geography in schools.

In addition, a total of 41% stated that YouTube was able to improve their English writing skills. Therefore, the use of YouTube applications in teaching and learning (T&L) helps a lot in writing, grammar and speaking skills. However, based on the overall analysis, it can be concluded that the YouTube application is more suitable to be used to master speaking skills. Apart from that, the use of Slideshare application among trainee teachers also clearly showed a medium level with a mean value of 2.64 and a standard deviation of 0.502. A study by Baharuddin and Badusah (2016) also showed that the level of use of Slideshare application was at a low level with a mean value of 1.93 and a standard deviation of 0.831.

Besides, the use of Web 2.0 and Internet applications helps trainee teachers in facilitating teaching and learning (T&L). This study is in line with a study conducted by Andin et al. (2014) on teachers from six schools in Manjung District around the Remis Beruas Coastal Zone. The results of the study showed that 90.8% of teachers stated that the use of ICT had facilitated teaching and learning (T&L). Thus, the results of this study also showed that trainee teachers used Web 2.0 and Internet applications uploading and downloading T&L materials easily and that was interesting to students.

In addition to that, the use of Web 2.0 and the Internet can also improve knowledge and skills in computer literacy. This is because the mastery of computer knowledge and skills among teachers is a strength for the use of technology in teaching and learning as well as a teaching experience for the teachers (Habibah and Vasugiammai, 2011). Thus, these skills and knowledge are needed so that educators can properly adapt or use Web 2.0 applications in the learning process.

### 5.2 Assessment on the effectiveness of using Web 2.0 applications between geography teachers and other teachers in global context

Overall, the analysis of the level of effectiveness of Web 2.0 and Internet use among trainee teachers showed a high level with an overall mean score of 4.45 and a standard deviation of 0.623. Based on Table 7, the highest mean is on *“textbook content, library and knowledge of trainee teachers can be enhanced with the new approach of using Web 2.0 applications”* with a mean score of 4.55 and a standard deviation of 0.557. Meanwhile, the lowest mean score is on *“Internet use can accommodate various needs of students separately through the learning and teaching (T&L) method of trainee teachers”* with a mean score of 4.29 and standard deviation of 0.700.

Based on the analysis of the effectiveness of Web 2.0 and Internet use, most trainee teachers showed a positive level on the use of ICT in the teaching and learning of Geography in schools. This is also in line with Kee-Man (2013) who supports the use of Web 2.0 applications in informal teaching and learning (T&L) to students. Based on the researcher's analysis, the use of social media such as Wikipedia, YouTube, Facebook and Twitter were found to be helpful in the sharing of knowledge and information openly as well as creating social interaction among students. This study also showed that the statement *“the use of Facebook, blogs, video sharing (YT), Twitter and Slideshare can encourage online learning”* was also at a high level with a mean of 4.53 and a standard deviation of 0.593. Thus, the use of Web 2.0 and the Internet is undeniably able to lead to a new teaching and learning process in 21st Century Learning.

Following that, a study conducted by Kee-Man (2013) on YouTube could help in improving speaking proficiency skills. The study presented undergraduate students' perceptions of the use of YouTube in English language learning provided in e-learning. The analysis found that 75% of students agreed and 25% of students disagreed that YouTube helped a lot in speaking skills. Watching videos on YouTube helped students a lot with correct and effective pronunciation and pronunciation techniques. However, there were also 45% of students agreed that YouTube could help them understand grammar. This can also be seen in a study by Ying Mai and Sanghee (2014) on the use of social media among nurses in the twelve states involved in the United States (US). The results of the analysis showed that Slideshare (n = 38, 9.92%) was a social media application that was rarely used by nurses compared to other social media. Therefore, the Slideshare application is not suitable to be used in teaching and learning (T&L).

In addition, the use of Prezi application in teaching and learning (T&L) can add more experience in using Prezi application. A study conducted by Duffy et al. (2015) showed that the use of the Prezi application was very beneficial for students' learning. The analysis done by the researchers showed that the majority, that is 98.6%, found that Prezi gave them an interesting experience compared to other ways of conducting Teaching and learning (T&L). The researchers' analysis also stated that 89.2% of the students provided a useful overview of the use of the Prezi application. However, only 31.1% of students had problems when using Prezi and all of them were technical issues.

Furthermore, according to a study conducted by Wafaa (2015), Prezi could help improve vocabulary among students. The study conducted by the researcher covered the effects of using Prezi on the vocabulary of second-grade female students in the fifteenth High School in Al-Madinah Al-Munawarah City, Saudi Arabia. The researcher conducted a T-Test study on 66 female students. The researcher's analysis showed that there was a difference in vocabulary achievement between using Prezi and not using Prezi. The results showed that the vocabulary teaching by using Prezi for female students recorded a mean of 20.34 and a standard deviation of 5.48 compared to the teaching without using Prezi that recorded a mean of 16.88 and a standard deviation of 4.71. Therefore, teaching and learning (T&L) by using the Prezi application can help students understand vocabulary more effectively.

Apart from that, the use of the Facebook application can contribute to the development system of formal and informal education. Other than using Facebook to socialize, users can indirectly use Facebook as an academic medium. According to Selwyn (2009), it is shown that the Facebook application is used for sharing, discussion, experience, assessment and moral support towards e-Learning. Meanwhile, a study has been conducted by Kee-Man (2013) on students' perceptions of the use of social media applications in English teaching. The researcher aimed to see the effectiveness of the Facebook application in the T&L of the English language. The overall analysis shows that Facebook was able to enhance scientific and social discussion. Based on the data obtained, it was found that 85% agreed that they discussed via Facebook media and 83% agreed that they liked to discuss via Facebook. And only 67% of them agreed to take the opportunity to be actively involved in the media. This can also be seen in the results of a study conducted by Roblyer et al. (2010) showing that most students preferred to use the Facebook application as a more effective medium for discussion and sharing.

## 6 Conclusion and limitation

Overall, this study has discussed the level of knowledge, skills and attitude, level of use of Web 2.0 applications and level of assessment on the effectiveness of Web 2.0 application and Internet use in teaching and learning (T&L) of Geography subject among Geography education program trainee teachers compared to other teachers in Malaysia and the worldwide basis. With this regard, it is a priority for all parties, especially graduate students, educators and educational information technology development to continue in strengthening the use of information and communication technology in all fields. This is because teachers are the people closest to the students in the school. Therefore, teachers should have a sufficient capacity in understanding the basic needs and demands of the current learning process mainly in digital age. As a result, teachers need to be given autonomy so that they can create a 21st century learning environment. Therefore, this will indirectly provide room and opportunity for teachers to explore ideas to build an effective and conducive environment for T&L. Since, the current study only focus on the context of one university in Malaysia, then the further research could be employed among school teachers who are experienced in teaching geography subjects with readiness, preparedness and challenges in conducting T&L by using various VLE applications.

## Declarations

### Author contribution statement

Miftachul Huda: Contributed reagents, materials, analysis tools or data; Wrote the paper.

Fauziah Che Leh: Conceived and designed the experiments.

Arnold Anduroh: Performed the experiments; Analyzed and interpreted the data.

### Funding statement

This research did not receive any specific grant from funding agencies in the public, commercial, or not-for-profit sectors.

### Data availability statement

Data included in article/supplementary material/referenced in article.

### Declaration of interests statement

The authors declare no conflict of interest.

### Additional information

No additional information is available for this paper.
